# Supplementary material for: Statistical Considerations for the Design and Analysis of Pragmatic Trials in Aging Research
Source: Geriatrics (Basel). 2024 Jun 4;9(3):75. doi: 10.3390/geriatrics9030075 (PMC11203240; doi:10.3390/geriatrics9030075)
Supplement: Supplementary file 1 [file geriatrics-09-00075-s001.zip › geriatrics-3022824-supplementary.pdf]

Supplementary Table S1. Two hypothetical trials in aging research scored according to PRECIS-2 for their explanatory/pragmatic design characteristics.

Domain	Trial 1	Trial 2	Rationale
	Score		
**Eligibility**	**2**	5	In Trial 1, a score of 2 was assigned because it is a clinical trial for a new drug, and therefore, it aims to exclude patients at higher risk of adverse events, leading to more stringent selection criteria. Conversely, in Trial 2, the score was 5. In this scenario, the policymaker seeks to maximize the benefits for the elderly population within the jurisdiction, resulting in more lenient selection criteria.
Recruitment	1	5	In the case of Trial 1, a score of 1 was assigned because multiple efforts will be made during patient recruitment. This is because it involves a selected population of elderly adults, and the selection criteria were somewhat strict, so multiple strategies will be implemented to recruit participants. In contrast, for Trial 2, a score of 5 was assigned, because the intervention will be provided to all those who receive care on a routine basis at the jurisdiction's clinics, which will facilitate participant recruitment.
Setting	1	5	For Trial 1, a score of 1 was assigned because it will be conducted in a single center, in a controlled environment, to constantly assess the safety of the treatment. This will require well-equipped facilities, so it will be conducted at an academic center where the investigator works. For Trial 2, on the other hand, a score of 5 was assigned because it will be conducted in the setting where patients receive their usual care.
Organization	1	5	In Trial 1, a score of 1 was assigned because, being a study of a new drug, it is necessary to have highly trained and certified personnel. On the other hand, for Trial 2, since it will be conducted where patients receive their usual treatment, infrastructures different from the usual ones are not expected; hence, a score of 5 was assigned.
Flexibility (delivery)	1	5	In the case of Trial 1, a score of 1 was assigned, because it is desired that the protocol be followed strictly, which is why measures will be taken to increase patient compliance. Conversely, a score of 5 was assigned for Trial 2, where such measures will not be implemented, allowing for flexibility in usual care.
Flexibility (adherence)	2	4	For Trial 1, a score of 2 was assigned since, in this case, to preserve the benefits of randomization over treatment groups, this study will not exclude participants after randomization. Instead, other measures will be encouraged to promote patient adherence to the intervention. On the contrary, for Trial 2, a score of 4 was assigned because no patients will be excluded after randomization. Additionally, not many measures will be implemented to increase adherence, but follow-up will be done by occasional phone calls.
Follow-up	1	3	In the case of Trial 1, exclusive visits will be conducted for the evaluation of laboratory studies and adverse events, as study intentions. Therefore, a score of 1 was assigned. In Trial 2, a score of 3 was assigned because patients will not undergo the usual follow-up. Instead, there is an intention to evaluate secondary outcomes to take advantage of the clinical trial infrastructure with a group of interested researchers; therefore, occasional visits will be implemented.

Primary outcome	2	5	For Trial 1, due to its high cost associated with evaluating the drug's safety and efficacy, resources for long-term follow-up, which would be desirable, are not available. Therefore, a change in an advanced coronary artery imaging study will be used as the primary outcome. Despite leaning more towards the explanatory spectrum, the knowledge of whether there is a change or deterioration in cardiac function may be relevant to the patient. Therefore, a score of 2 was assigned. In contrast, Trial 2 was assigned a score of 5 because it will assess, in the long term, those who will develop secondary cardiovascular events versus those who will not. Therefore, this outcome holds great importance and relevance for patients.
Primary analysis	3	5	In Trial 1, a score of 3 was assigned because, as mentioned earlier, patients will not be excluded with the intention of preserving the benefits of randomization. Therefore, an intention-to-treat analysis will be conducted, but there is also interest in performing a per-protocol analysis. Since both analyses are contemplated, a score of 3 is assigned. Lastly, Trial 2 has a score of 5 because in this case an intention-to-treat analysis is intended without excluding patients after randomization.
**Average score**	**1.6**	**4.7**	
